# Comparison of prepartum serum concentration of vitamin E and selenium from dairy cows within herds with high and low incidence of retained placenta

**DOI:** 10.3168/jdsc.2025-0933

**Published:** 2026-01-16

**Authors:** Nicolas Barbeau-Grégoire, Younès Chorfi, Maxime Leduc, Marianne Villettaz Robichaud, Jocelyn Dubuc

**Affiliations:** 1 Département de Sciences Cliniques, Faculté de Médecine Vétérinaire, Université de Montréal, St-Hyacinthe, Québec J2S 2M2, Canada; 2 My Forage System, Montréal, QC H1W 3J5, Canada

## Abstract

The goal of this observational study was to compare the prepartum vitamin E and selenium concentrations of multiparous cows based on herd incidence of retained placenta membranes (RP). Within a database of 50 herds enrolled by convenience for a previous study and followed for a whole year, 5 herds with no incidence of RP and 5 herds with high incidence (≥6.5%) of RP were randomly selected. In each farm, 10 multiparous cows were randomly selected. Vitamin E and selenium were analyzed by HPLC in serum collected from 21 to 7 d before predicted calving. No significant difference was observed in the concentration of vitamin E or selenium between cows from herds with no incidence of RP and herds with high incidence of RP. Our results suggest that prepartum concentrations of vitamin E and selenium are not good indicators of herd RP incidence status, and perhaps other predictors should be explored.

## Highlights

• Prepartum vitamin E and selenium were compared between herd RP incidence groups.
• Serum samples were collected 21 to 7 days before predicted calving.
• No difference was observed in vitamin E or selenium between herd RP groups.
• Micronutrients alone are not associated with herd RP incidence.

## Graphical Abstract

**Figure:**
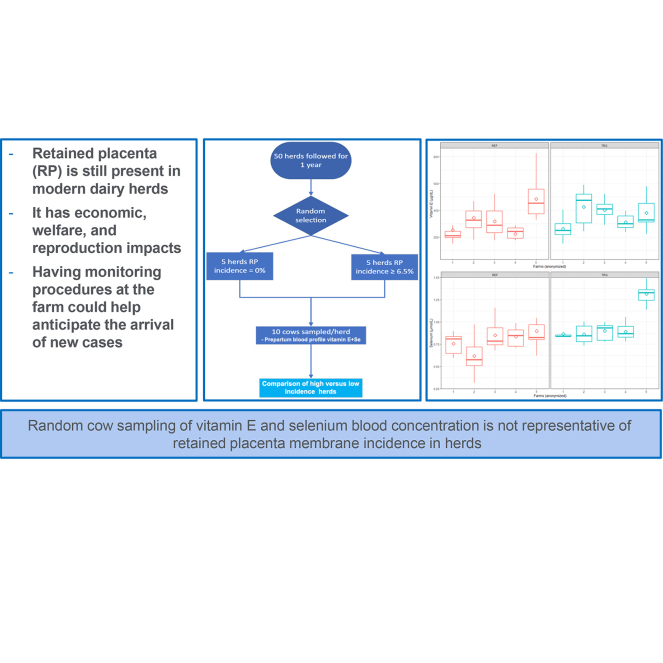
**Summary:** This observational study evaluated whether prepartum vitamin E and selenium concentrations in multiparous dairy cows were associated with herd incidence of retained placenta (RP). Ten dairy herds were selected from a larger database of 50 herds previously followed for 1 year: 5 herds with no RP cases and 5 herds with high RP incidence (≥6.5%). In each herd, serum samples were collected from 10 multiparous cows between 21 and 7 days before the predicted calving date. Vitamin E and selenium concentrations were measured using high-performance liquid chromatography. No significant differences in prepartum vitamin E or selenium concentrations were detected between cows from herds with no RP incidence and those from herds with high RP incidence. These findings suggest that prepartum vitamin E and selenium status alone may not adequately explain differences in herd-level RP incidence, and that other nutritional, metabolic, or management-related risk factors should be investigated.

Retained placenta (**RP**) is a metabolic disorder that is defined as the incapacity to expel the fetal membranes within 24 h following calving (Markusfeld, 1987). Retained placenta is a well-known disorder in modern dairy farming, and multiple research projects have focused on it (LeBlanc, 2008). Retained placenta increases the risk of metritis, displaced abomasum, and hyperketonemia (Han and Kim, 2005). In terms of cow performance, RP increases the number of days open and lowers milk production (Mahnani et al., 2021). The principal physiologic cause of RP is a malfunction in the separation between cotyledons and caruncles (Laven and Peters, 1996). These attachment points between the uterine wall and the placenta are normally dissolved by the actions of neutrophils (Horst et al., 2021). It is known that during the peripartum period, the immune system response (including neutrophils) is reduced due to negative energy balance status, high demand for calcium for milk production, and a drop in the blood concentration of vitamin E and selenium. Injections of vitamin E and selenium before calving have been successful in reducing the number of cases of RP on farms (Bourne et al., 2007). Vitamin E plays an important role in chemotaxis responsiveness and the cell membrane integrity of neutrophils, and selenium is essential to the synthesis of proteins with antioxidant capabilities, which affect neutrophil effectiveness (Ndiweni and Finch, 1996).

The blood metabolic profile is an interesting tool for veterinarians to help evaluate the risk of metabolic disorders. Combined with other parameters, it can help to target the right plan of actions to limit or prevent the disorders' impact. Although most major metabolites can be analyzed in sequential protocols, vitamin E and selenium need to be analyzed with specific techniques, which means more time per sample and higher costs. Pooled blood sampling could be used to save on costs and time for data analysis. Thus, analysis would be interpreted on the herd level. In preventive medicine, herd-level indicators are often used to target factors that have substantial effects on the herd as a whole. Those indicators are often more practical to apply on commercial farms because they are less time consuming and cheaper than individual analysis (Bouchard, 2021). To our knowledge, no research has compared dairy cows' blood concentrations of vitamin E and selenium based on RP incidence at the herd level.

The objective of this project is therefore to compare the prepartum vitamin E and selenium profiles of cows within herds having high and low incidence of RP. We hypothesized that multiparous cows in herds with a high incidence of RP would have lower blood concentrations of vitamin E and selenium compared with multiparous cows in herds with low incidence of RP.

The blood samples used in this project were collected from a database created for another observational study completed in 2023. The study was approved by the Animal Use Ethics Committee of the Université de Montréal (24-Rech-2179-2). Briefly, cows were enrolled from 50 commercial farms in the region of Saint-Hyacinthe, Québec, Canada, based on convenience criteria. The final sample group was composed of both tiestall and freestall farms and with different feeding strategies (mainly total mixed rations based on hay and corn silage). For 12 mo, monthly visits were completed on each farm, during which 2 cows meeting the following selection criteria were randomly selected: Holstein breed, between 21 and 7 d before expecting calving date, and multiparous. The herds' status for RP incidence for the whole year of collection was evaluated based on disorder recording in the farming management software used at the farm by producers and veterinarians. Farms with 0% of incidence of RP were grouped as the reference group (**REF**), and farms with an incidence of 6.5% or higher were grouped as target (**TRG**). These thresholds were used based on a performance report from dairy farms in Québec (Canada) on disease incidence (DSAHR, Saint-Hyacinthe, QC, Canada, personal communication, 2024). In each group, 5 farms were randomly selected (n = 10 total). From each of those farms, 10 cows were randomly selected within the 24 cows enrolled in the initial project (n = 100 cows total). On the cow level, no consideration of RP incidence was given in the selection of individuals.

Blood samples were collected from the coccygeal vein and into dry tubes without anticoagulant (Vacutainer, Becton Dickinson and Company) and immediately placed into a covered ice bucket to protect the samples from heat and ambient light. Within 4 h of collection, the samples were centrifuged for 15 min at 2,000 × *g* at 22°C (room temperature). The serum was removed and stored in a cryotube at −80°C until the time of analysis. Vitamin E was analyzed by reverse-phase HPLC based on the method published by Gueguen et al. (2002) and adapted by our laboratory. Selenium was measured by a modified HPLC method reported by Hawkes and Kutnink (1996). Conditions of compliance criteria and quality control included coefficient of determination of standard curve >0.98, relative standard deviation ≤20%, limit of detection = 85% to 115%, and quantification limit = 80% to 120%.

Statistics analyses were completed using R 4.4.2 (R Foundation for Statistical Computing) software (lme4 package; Bates et al., 2015). Cow was the experimental unit, which could either be in a farm with high or low incidence of RP. For each of the farm groups, descriptive statistics of vitamin E and selenium concentrations were computed. Mixed linear models were used to assess the effect of RP herd status on concentrations of both metabolites. The clustered structure of the data (herds) was considered by implying a random effect to the models.

The comparison of vitamin E serum concentration of cows from herds with high and low incidence of RP is shown in Figure 1. Retained placenta incidence did not have a significant effect on vitamin E concentration (*P* = 0.57). A herd effect was notable, with an intraclass correlation coefficient (**ICC**) of 0.33, suggesting that a third of the vitamin E concentration was explained by farm-to-farm difference.

**Figure 1:**
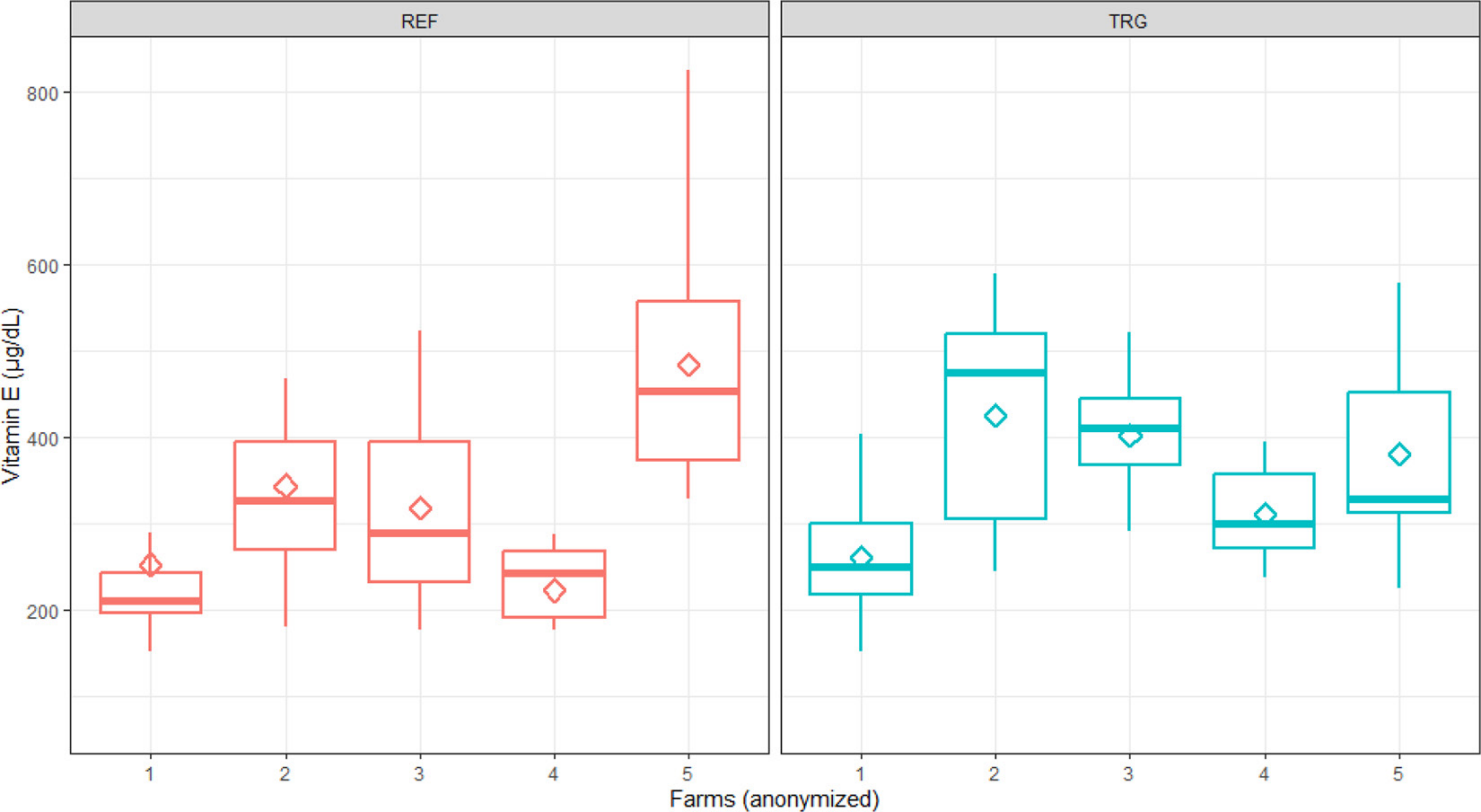
Comparison of prepartum vitamin E serum concentrations from multiparous Holstein cows within herds with low (reference [REF], 0% incidence) and high incidence (target [TRG], incidence of 6.5% or higher) of RP. Ten cows were analyzed within each farm. The diamond represents the mean, and the midline is the median. The diamond represents the mean, the midline is the median, and the lower and upper edges, respectively, are the 25th and 75th percentile. Whiskers extend to 1.5 × the interquartile range.

The comparison of selenium serum concentrations of cows from herds with high and low incidence of RP is shown in Figure 2. Retained placenta incidence did not have a significant effect on selenium concentration (*P* > 0.25). The ICC of the herd effect on selenium concentration was 0.25, suggesting that an important part of selenium variation was explained by farm-to-farm differences.

**Figure 2:**
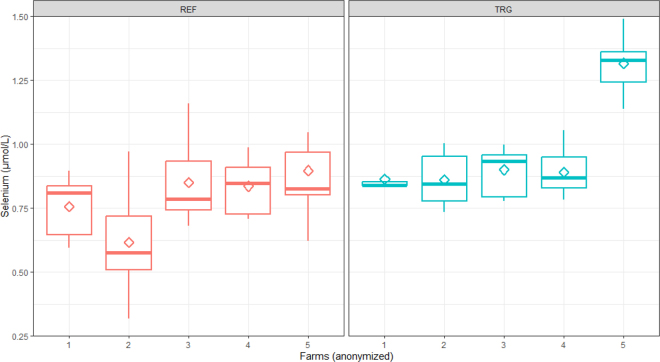
Comparison of prepartum selenium serum concentrations from multiparous Holstein cows within herds with low (reference [REF], 0% incidence) and high incidence (target [TRG], incidence of 6.5% or higher) of RP. Ten cows were analyzed within each farm. The diamond represents the mean, and the midline is the median.

The goal of this project was to compare prepartum vitamin E and selenium serum concentrations of multiparous cows from farms with high and low incidence of RP. In a preventive medicine approach, it is important to use health indicators that can be easily monitored on a regular basis and that have been linked with the condition of interest. At the cow level, the effects of vitamin E and selenium on dairy cow metabolism are well known. However, techniques used to estimate concentrations of those 2 metabolites are costly and are generally only used clinically when an increased in incidence of RP is observed on-farm in order to investigate the source of the problem. Little is known about the predictive potential of those 2 metabolites at the herd level. Therefore, a pooled-based approach for blood analysis of vitamin E and selenium could be a more time- and cost-effective method to use in preventive medicine. Given this context, examining the variation in blood concentrations of these 2 metabolites based on herd RP status was a logical first step. Our results showed no significant difference in the blood concentrations of vitamin E and selenium in multiparous Holstein cows from herds with high incidence of RP compared with those from herds with no incidence of RP. If we consider the data purely numerically, both metabolites were slightly higher in herds with RP. These results are in contrast with our knowledge of the importance of vitamin E and selenium in the normal process for liberation of placenta membranes in dairy cows. This could be explained by the fact that we did not control for any on-farm protocols that could affect vitamin E or selenium concentrations (injections, additives). Therefore, it is plausible that farms with high RP incidence, acknowledging its negative effects on production, had already put in place strategies to ensure the recommended blood concentrations of vitamin E and selenium in prepartum cows were met. Sampling earlier in the dry-off period could have better reflected the nutritional status of vitamin E and selenium and led to obtaining different results. Because retained placenta is a metabolite disorder, it can be caused by multiple combined factors, such as milk production, energy balance, dystocia, calcium management, and preparation feeding strategies. Those factors were not controlled in this project and perhaps could have been meaningful in explaining the high incidence in our target group. Our results support this idea that RP is multifactorial, and thus controlling one risk factor (vitamin E and selenium) might not be sufficient to limit on-farm RP incidence. Cows that were selected to create our sample in both groups were randomly selected within each herd without knowing how many of them have had a case of RP postpartum. Our results might have been more aligned with what was expected if we had included a selection criterion based on individual RP status. However, that would have been against the main goal of this project, which was to find relations between herd RP incidence and random on-farm sampling of multiparous individuals. Our results do not support our initial hypothesis that prepartum blood concentrations of vitamin E and selenium could be used as an indicator of RP risk factors at the herd level.

Our project did not have high statistical power because of the low number of cows enrolled (n = 100). Financial resources limited our number of individuals analyzed. This study was exploratory and aimed to determine if vitamin E and selenium could be useful predictors in our main research in a way that could be practical and cost effective on-farm. Nonetheless, we had multiple herds within each group (n = 5), limiting the impact of other factors within a farm. Our results suggest that random pooled analysis of prepartum vitamin E and selenium serum concentrations are not good indicators of the herd RP incidence status. Research with more statistical power could be completed to confirm our results. Other factors known to be risk factors of RP should be explored to find some that could be associated with RP incidence at the herd level. Herd-level studies are challenging because of the need to find multiple herds to enroll, but they bring very informative data on a practical application point of view. Because RP is a condition with a high impact on cows and is still present in modern dairy farms, more studies should be focused on finding predictive indicators of a high risk of RP.

In conclusion, the results in this study suggest that the serum concentration of either vitamin E or selenium from cows randomly sampled 21 to 7 d prior to the predicted calving date does not reflect farm incidence of RP. Considering the negative impact RP has on dairy farms, further studies are needed to find potential predictive indicators at the herd level.
